# Scientific Rationale for the Use of Alpha-Adrenergic Agonists and Glucocorticoids in the Therapy of Pediatric Stridor

**DOI:** 10.1155/2011/575018

**Published:** 2011-12-19

**Authors:** Gustavo Nino, Orkun Baloglu, Maria J. Gutierrez, Michael Schwartz

**Affiliations:** ^1^Division of Pediatric Pulmonary Medicine, Penn State Hershey Children's Hospital, Pennsylvania State University College of Medicine, Hershey, PA 17033, USA; ^3^Division of Allergy and Immunology, Pennsylvania State University College of Medicine, Hershey, PA 17033, USA; ^2^Penn State Sleep Research and Treatment Center, Pennsylvania State University College of Medicine, 500 University Drive, Hershey, PA 17033, USA

## Abstract

*Purpose*. The most common pharmacological therapies used in the treatment of stridor in children are glucocorticosteroids (GC) and alpha-adrenergic (*α*AR) agonists. Despite the long-standing reported efficacy of these medications, there is a paucity of data relating to their actual mechanisms of action in the upper airway. *Summary*. There is compelling scientific evidence supporting the use of *α*AR-agonists and GCs in pediatric stridor. *α*AR signaling and GCs regulate the vasomotor tone in the upper airway mucosa. The latter translates into better airflow dynamics, as delineated by human and nonhuman upper airway physiological models. In turn, clinical trials have demonstrated that GCs and the nonselective *α*AR agonist, epinephrine, improve respiratory distress scores and reduce the need for further medical care in children with stridor. Future research is needed to investigate the role of selective *α*AR agonists and the potential synergism of GCs and *α*AR-signaling in the treatment of upper airway obstruction and stridor.

## 1. Introduction

Stridor is a common presenting symptom in children, and its treatment must be tailored according to its underlying condition. Even though there are many disorders that can induce stridor, they often share a common pathophysiology producing dynamic collapse of the extrathoracic airway during inspiration [1, 2]. This resultant inspiratory flow limitation may be associated with a range of impairment, producing increasing breathing efforts and distress. When surgical correction is not indicated, for instance when stridor is caused by viral croup or occurs immediately after extubation, pharmacological agents are primarily used. The main goal of pharmacological therapy for stridor is to alleviate, or prevent, upper airway collapse during inspiration by optimizing intraluminal patency and airway wall stability. The most common pharmacological therapies used in children are glucocorticosteroids (GC) and alpha-adrenergic (*α*AR) agonists, particularly dexamethasone and inhaled epinephrine, respectively [3]. Despite the long-standing reported efficacy of these medications, there is a paucity of data describing their actual mechanisms of action in pediatric stridor. This article aims to review the current knowledge of the physiological activity of *α*AR agonists and GCs in the upper airway, as well as the potential synergistic effect of their combined administration. Also reviewed is the current clinical evidence describing the safety and efficacy of these medications for the treatment of stridor in children.

## 2. Alpha-Adrenergic (***α***AR) Agonists and the Regulation of the Vasomotor Tone of the Upper Airway

### 2.1. Molecular and Cellular Effects of ***α***AR Signaling in the Upper Airway

Alpha-adrenoreceptor (*α*AR) signaling has a significant regulatory role in maintaining the vasomotor tone of the upper airway [4–8]. The *α*1 and *α*2 AR are located in the smooth muscle (SM) of the nasal cavernous bodies and the vascular framework of the mucosa that covers the entire upper airway [4–13]. The stimulation of these receptors enhances SM contractile tone, which in turn increases nasal [4, 8] and oropharyngeal patency [5, 6]. The primary cellular mechanism elicited by *α*AR-stimulation in SM is G-protein coupled receptor-(GPCR-) mediated activation of intracellular cascades that upregulate calcium-mediated SM contraction [14, 15] and ameliorate cAMP-induced SM relaxation [13, 16]. The physiological result of this *α*AR-induced SM contractile tone is a decreased thickness of the mucosa and other soft tissue layers, thereby increasing the intraluminal space of the upper airway. In this regard, the thickness of the mucosa in the airway is largely determined by (1) blood flow regulated by the mucosal vascular tone and (2) interstitial fluid collection, or edema, which is the end-product of an imbalance between interstitial-oncotic forces, vascular permeability, and lymphatic efflux [17, 18]. Wasicko et al. demonstrated that *α*AR-agonists exert their primary effect in the upper airway mucosal mass by regulating the intravascular blood flow [5]. This was supported by magnetic resonance imaging (MRI) of the upper airway of animal models that showed immediate changes in the upper airway mucosal size and tissue water content in response to the infusion with the *α*AR-agonist vasoconstrictive agent phenylephrine [5]. These changes were completely reversed by the vasodilating agent sodium nitroprusside, which led the authors to conclude that the *α*AR-induced changes in mucosal size are unlikely to be caused by variations in the interstitial fluid volume. The later findings are further substantiated by experiments demonstrating rapid and reversible fluctuations in the presence of the intravascular contrast agent Gd-DTPA in the upper airway mucosa following phenylephrine and sodium nitroprusside administration [5]. In addition, topical administration of *α*AR-agonists induces rapid and dramatic changes in the size of the nasal mucosa [6, 8, 19] and oropharyngeal mucosa of human subjects [6]. Taken together, the clinical implication of these findings is that the pharmacological action of *α*AR-agonists involves a profound effect in the mucosal size (vascular tone) and airway patency (Table 1), but that this therapy does not target the underlying disease mechanism in many instances, such as with airway edema. Thus, the beneficial effects of *α*AR-agonists on mucosal mass size and airflow dynamics are likely to be transient.

### 2.2. *α*AR-Agonist Mediated Effects in Nasal and Oropharyngeal Airflow Dynamics

The previously described cellular and molecular effects of *α*AR-stimulation in upper airway mucosa have raised an important question: do these changes in the airway mucosal size and patency translate into beneficial mechanical changes in airway wall collapsibility and airflow? This concept is important because the majority of pediatric conditions associated with stridor and upper airway obstruction are characterized by dynamic inspiratory collapse of the upper airway [1–3]. Indeed, there is compelling evidence demonstrating that *α*AR-agonists enhance both nasal cavity cross-sectional area and nasal airflow, assessed by acoustic rhinometry (AR) [19, 20] and peak nasal inspiratory flow (PNIF), respectively [21]. Interestingly, Kjaergaard et al. have recently reported that changes in nasal cavity geometry induced by *α*AR-agonists directly correlates with increased nasal airflow in a linear fashion [22]. This finding is probably explained by the active action of the alae nasi muscles that help prevent nasal collapse even under conditions of maximal nasal inspiratory flow [23]. 

*α*AR-agonists also improve airflow mechanics in the oropharyngeal airway. Experiments using *in vivo* animal models of sealed upper airways have demonstrated that the closing and opening pressures of the oropharynx are transiently modified by the administration of phenylephrine [5]. In addition, topical oropharyngeal phenylephrine use in humans causes decreased airflow resistance independently of changes in nasal resistance or upper airway muscle tone [6]. These effects of *α*AR-signaling in the regulation of oropharyngeal airflow and collapsibility are attributed to the preferential action of *α*AR-agonists in the posterolateral mucosa of the oropharynx [5], an area prone to collapse under conditions of upper airway obstruction [24, 25]. In conclusion, the pharmacological effects of *α*AR-agonists in the treatment of stridor and upper airway obstruction seem to be related to their regulatory role and increasing vasomotor tone of the nasal and posterolateral oropharyngeal airway mucosa. The consequent effects of this increased vasomotor tone are likely changes in the mechanical properties of the upper airway resulting in less collapsibility, less resistance, and improved airflow (Table 2).

## 3. Glucocorticosteroid (GC) Effects on the Upper Airway Function

### 3.1. Molecular and Cellular Effects of GC Signaling

Glucocorticosteroids (GCs) are hormones with a ubiquitous intracellular receptor (GR) and a broad range of biological actions in different body cells and systems. The primary cellular mechanism elicited by GR-activation is the stimulation of the GC response elements (GREs) in the promoter region of primary or secondary genes [26]. The GRE complexes modulate transcription factors such as NF-*κ*B or AP-1 which are considered critical for many proinflammatory signals [26, 27]. Additional mechanisms include the regulation of intracellular mitogen-activated protein kinases (MAPKs) [28–30] and nongenomic GC-mediated actions that include the regulation of second messengers and ion channels [31]. The most common therapeutic use of GCs is the attenuation of inflammation and immune responses via GR activation in T-cell lymphocytes and other immune cells [26, 32, 33]. However, GCs are also known to modulate the function of multiple components in the respiratory system including cells in the epithelium [34, 35] as well as smooth muscle in the airways [29, 34, 36, 37] and blood vessels [38, 39]. Accordingly, GCs appear to exert their action in the upper airway through the regulation of several processes in different cell lines.

### 3.2. Genomic and Nongenomic Mechanisms of Action of GC in the Upper Airway

GCs typically exert rapid, delayed, and long-term effects in the upper airway [40]. Long-term effects are likely due to GRE-mediated antiinflammatory action and suppressed microvascular permeability yielding less mucosal edema formation [40]. There is compelling evidence demonstrating that topical GC in patients with allergic rhinitis and other types of upper airway inflammation significantly reduces the local release of multiple cytokines and vasoactive inflammatory mediators [40–43], and also lessens cellular infiltration in the respiratory mucosa [44]. Moreover, GCs modulate the expression of vascular endothelial growth factor (VEGF), which is a crucial angiogenesis stimulator that induces vascular permeability and vasodilatation via nitrogen oxide (NO) synthesis by the endothelium [45, 46]. 

In addition to GRE-mediated genomic mechanisms, there are also nongenomic actions of GCs that can potentially mediate more rapid effects of GC in upper airway function. For instance, GCs inhibit the synthesis of arachidonic acid derivatives that affect vascular permeability and tone (i.e., prostaglandins) [47]. GCs also affect the vascular smooth muscle tone by directly regulating intracellular levels of calcium and relaxant cyclic nucleotides (cAMP and cGMP) [29, 48] and indirectly enhancing *α*AR-mediated signaling [49]. Another nongenomic action of GC is the regulation of Cl-ion channels and Na^+^/H^+^ exchange in respiratory epithelial cells, which induces an immediate decrease in net water efflux [50–52]. As a result, nongenomic effects of GCs can potentially induce rapid volume changes in the intravascular and interstitial compartments of the airway mucosa reducing the thickness of the airway wall [53–55]. 

These cellular and molecular mechanisms of GC action translate in a reduction of upper airway flow obstruction and stridor [56, 57] with a delayed peak of clinical effect of approximately 6 hours after their initial administration [57]. Taken together, this evidence suggests that a combination of genomic and nongenomic actions of GCs is necessary for the full pharmacological activity of GCs on upper airflow dynamics (Tables 1 and 2).

## 4. Molecular Interactions of ***α***AR Agonists and GC in the Upper Airway: Modulating ***α***-AR Homologous Desensitization

### 4.1. *α*AR Agonists-Induced Homologous Desensitization in the Upper Airway

Although *α*AR agonists acutely relieve upper airway obstruction [58], their chronic use induces desensitization to the *α*AR-mediated effect (tachyphylaxis) and paradoxical rebound obstruction [59–62]. This is supported by the increased upper airway resistance observed in healthy subjects who have received prolonged *α*AR-agonist administration [59]. In addition, chronic nasal administration of nonselective *α*1/*α*2 agonists (i.e., oxymetazoline) induces *rhinitis medicamentosa*, a condition characterized by rebound nasal congestion and pathologic changes in the vasculature of the nasal mucosal [63]. This rebound effect observed following sustained *α*AR-stimulation can also occur after administration of other *α*AR agonists utilized in the therapy of upper airway obstruction and stridor [64]. This paradoxical effect of *α*AR-signaling is termed homologous desensitization, and it is observed following prolonged stimulation of all types of *α* and *β* adrenoreceptors [65–67] and other G-protein coupled receptors [68]. There are several molecular mechanisms that may explain this desensitization, including internalization and uncoupling of the surface AR [51] and modulation of the AR-coupled intracellular signaling pathways that regulate second messengers (such as cAMP, cGMP, IP3, and calcium) [65–68]. This phenomenon of AR-induced paradoxical rebound obstruction raises serious safety concerns regarding the sole use of these types of medications for the treatment of numerous upper airway conditions [69].

### 4.2. Beneficial Effects of GC in *α*AR Signaling and Homologous Desensitization

GCs prevent the generation of AR-homologous desensitization in different cell lines [29, 70, 71]. They increase the cell surface *α*AR and *β*AR numbers in the respiratory system [49, 72] as well as upregulate AR downstream signaling by modulating activation of protein kinases, including the MAPKs, ERK1/2, P38, and c-Jun NH2-terminal kinase (JNK) [29, 73]. As a result, GCs exert a synergistic action on AR agonist therapy for numerous airway diseases [74]. In the upper airway, GCs modulate the effects of *α*AR-agonists on the airway vasomotor tone, thereby improving airflow dynamics [56]. Topical GCs even reduce upper airway mucosal size in individuals with nonallergic vasomotor rhinitis [75–77] and prevent the development of *α*AR-agonist-induced tachyphylaxis [78]. Furthermore, recent randomized clinical studies have suggested that the combination of GC and *α*AR-agonists together provide a synergistic effect in the treatment of nasal [78, 79] and more distal airway obstruction [80]. This evidence underscores the importance of the nonimmune-mediated mechanisms of action of GC in the regulation of the vasomotor tone and upper airway function. 

In concert with the beneficial effects of GCs in *α*AR-signaling, the use of nonselective *α*AR agonists with *β*AR activity for the treatment of stridor (i.e., epinephrine) may lead to enhanced GC signaling via *β*2-AR-induced upregulation of GRE-dependent transcription [81]. In this regard, prior investigations have demonstrated that the addition of long-acting *β*2-AR agonists to GC therapy results in upexpression of various antiinflammatory proteins such as GC-inducible leucine zipper (GILZ) and MKP-1 to an extent not achievable by exposure to GC alone [81]. The latter evidence suggests that the antiinflammatory action of GCs in the upper airway might be potentially augmented by simultaneous administration of nonselective AR-agonists with *β*-AR activity such as epinephrine; however, this possibility remains to be systematically investigated.

**Figure 1:**
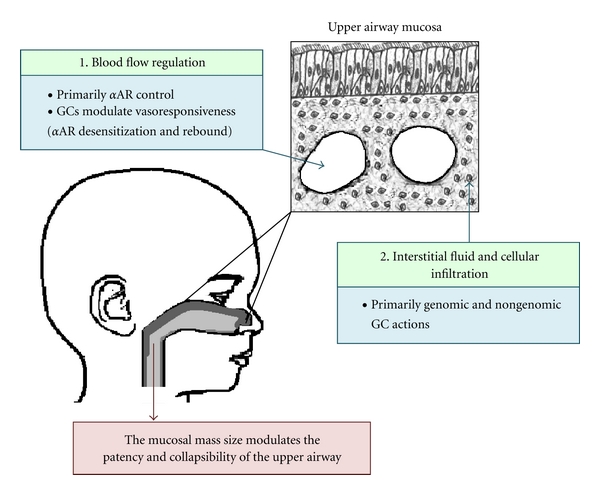
Regulatory effects of alpha-adrenoreceptor-(*α*AR-) agonists and glucocorticoids (GCs) in the upper airway mucosa.

In summary, there are several molecular interactions between *α*AR and GC signaling that influence upper airway flow dynamics (Figure 1). These positive interactions potentially provide a physiological rationale for the combined use of *α*AR-agonist and GCs in the therapy of upper airway obstruction and stridor.

## 5. Clinical Evidence of the Use of ***α***-AR Agonists and GC in the Treatment of Pediatric Stridor

Clinical conditions that produce new onset stridor in children are typically associated with increased mucosal thickness and resultant intraluminal narrowing of the extrathoracic airway (nose, pharynx, larynx, and trachea). There are two major mechanisms of disease causing upper airway mucosa swelling in children: (1) *infectious/inflammatory related*, most commonly associated with viral laryngotracheobronchitis, or “croup” and (2) *mechanical trauma*, most often related to endotracheal intubation and foreign bodies in the upper airway. The clinical evidence supporting the efficacy of *α*AR agonists and GCs in the treatment of both these types of stridor-causing mechanisms in children is discussed next. 

### 5.1. Role of *α*AR-Agonists in the Treatment of Viral-Induced Stridor

Randomized controlled trials (RCTs) have consistently demonstrated that the nebulized use of the nonselective *α*AR-agonist and *β*AR-agonist, epinephrine, significantly relieves the distressing symptoms of croup in young children [82–87]. Indeed, the effectiveness of epinephrine in the treatment of stridor associated with croup has been extensively studied in pediatric age group since the 1970s [82–85]. Many initial studies demonstrated significant improvements in croup symptom scores compared to placebo at 10 and 30 minutes after administration, with waning effects by 120 minutes after treatment [82–87]. In addition to changes in clinical scores, inhaled epinephrine significantly reduces tracheal diameter [88], and improves breathing mechanics [89] in children with stridor from viral croup.

Most initial clinical trials studying pediatric stridor focused on the efficacy of treatment with the racemic form of epinephrine, which contains dextro (R) and levo (L) isomers. Waisman et al. then established that nebulized isomeric (L) epinephrine, which is more widely available than the racemic form, is equally effective for the treatment of viral croup in children [86]. In addition, although inhaled epinephrine is the only *α*AR-agonist therapy with sufficient clinical evidence to support its use in pediatric viral-induced stridor [87], there is data suggesting that other *α*AR-agonists could be efficacious [64]. This knowledge is important because epinephrine, which is a catecholamine with nonselective *α*AR and *β*AR agonist properties, has significant cardiovascular side effects related to its poor receptor specificity [90]. In this regard, Lenney and Milner reported that a single inhalation with phenylephrine (a selective *α*AR-agonist) in a small cohort of young children with viral croup, produced transient clinical improvement of stridor and decreased respiratory resistance, measured by modified forced oscillation [64]. Moreover, a recent RCT demonstrated that the nasal administration of the pure *α*AR-agonist, xylometazoline, was equally effective compared to nebulized epinephrine in decreasing respiratory distress in the setting of a viral respiratory illness [91]. These findings are consistent with the prevailing concept that the adrenergic control of the upper airway is largely mediated by *α*AR-signaling [4–13], and it suggests that the effect of epinephrine in reducing viral-induced stridor is most likely mediated by its *α*AR-properties. Nevertheless, there remains insufficient evidence to support the routine use of alternate *α*AR-agonists for the treatment of viral-induced stridor in children. 

The efficacy of nebulized epinephrine has recently been highlighted in a Cochrane review that evaluated its use for viral-induced croup in children [87]. This review included 8 RCTs or quasi-RCTs with a total pool of 225 children diagnosed with croup in the ER or inpatient hospital setting. The primary outcome of these studies was croup score after treatment with nebulized epinephrine. Secondary outcomes included duration of intubation, length of hospitalization, number of return visits for croup, parental anxiety level, and medication side effects. The authors concluded that the use of nebulized epinephrine, either as racemic or L-isomeric preparation, is significantly associated with transient reduction of croup symptoms 30 minutes after treatment [87]. Taken together, the current clinical evidence supports the use of nebulized epinephrine as the first-line rescue therapy for children presenting with stridor and croup-related respiratory distress elicited by acute viral laryngotracheobronchitis (Table 3).

### 5.2. GCs and the Treatment of Viral Croup in Children

GCs are considered the mainstay of therapy for viral croup in children [3, 92]. As previously described, GCs reduce upper airway swelling which leads to significant improvement in croup symptoms. Their onset of action is about an hour after administration, which is delayed compared to that of inhaled epinephrine, and their peak effect is noted 6–12 hours after administration [57, 93]. GCs have been used in the treatment of children presenting to an ER with stridor caused by viral-induced croup in children for many years, and their efficacy has been extensively studied using multiple administration routes. A seminal RCT conducted by Bjornson et al. [92] evaluated the efficacy of oral dexamethasone in 720 children with mild croup (croup score ≤2 on the Westley croup scoring system). They found that a single dose of oral dexamethasone (0.6 mg/kg/dose, maximum 20 mg) resulted in a significantly quicker resolution of symptoms and improvement in croup score compared to placebo [92]. Additional studies have consistently shown that GCs improve croup symptoms scores and reduce hospital admissions [57, 92–94]. In addition, a recent cochrane review of 38 RCTs, with a total of 4299 children pooled, further confirmed that GCs are effective in improving croup score symptoms and decreasing the number of return visits, (re)admissions, and hospital length of stay [57]. 

Multiple studies have investigated different routes, doses, and types of GCs in order to identify their optimal use in the management of viral croup in children. Oral dexamethasone (0.6 mg/kg/dose) has been found to be as effective as intramuscular dexamethasone [94], and both are superior to nebulized dexamethasone in the treatment of mild and moderately severe croup in children [95]. Interestingly, high-dose nebulized budesonide (2–4 mg) seems to be equally effective as either oral or intramuscular dexamethasone in treatment of croup [96–98]. Nebulized budesonide may also play a role as adjunct therapy to dexamethasone in the management of mild-moderate croup in the outpatient setting [99], but it does not add benefit in the treatment of children hospitalized with croup [100]. Conversely, the administration of high-dose fluticasone (2,000 micrograms) via metered dose inhaler (MDI) and spacer did not provide any clinical benefit in children with viral croup in a small RCT with 17 patients [101]. This might suggest that the correct use of MDI inhalers results in much less medication deposition in the upper airway and these devices may not be indicated for the treatment of stridor. With regards to alternate dosing, oral dexamethasone dosed at 0.15 mg/kg may be equally effective as dosing at 0.6 mg/kg for the treatment of moderate to severe croup [102–105]. Regarding the use of different classes of GCs, oral betamethasone (0.4 mg/kg/dose) seems as efficacious as oral dexamethasone (0.6 mg/kg/dose) in children with mild to moderate croup [106]. Oral prednisolone (1 mg/kg/dose) also improves croup clinical scores [104, 107], but it may be less effective than dexamethasone in reducing the number of unscheduled representations to medical care [107]. However, a recent RCT did not observe significant differences between the use of prednisolone (1 mg/kg/dose) and dexamethasone (dosed at either 0.15 or 0.6 mg/kg) in the treatment of children with mild to moderate croup [104]. 

 In summary, there is compelling evidence demonstrating the effectiveness of using GCs in the treatment of viral-induced stridor in the pediatric population (Table 3). Multiple RCTs studying children with viral croup have consistently shown significant improvement of clinical scores, less return visits, and shorter hospital stay following GC administration. Oral, parenteral and nebulized GCs (but not via MDI with spacer) appear to provide similar clinical benefits. There are no major differences in clinical response when using alternate routes of GC administration, but the inhaled route may need high-doses to have comparable effects. Given that the vast majority of the pediatric clinical evidence currently available relates to the use of dexamethasone (dose 0.15 mg/kg to 0.6 mg/kg), this GC is most recommended for the treatment of viral-induced stridor in children.

### 5.3. Trauma-Related Stridor in Children: *α*AR-Agonists and GCs in the Postextubation Period

Post-extubation stridor is seen uncommonly in adults, but it often occurs in young children under 5 years of age, with a reported incidence of 1–4% [108]. The pathophysiology of this condition involves local mucosal and submucosal trauma caused by the endotracheal tube with resultant transient intraluminal subglottic narrowing due to airway edema [109]. Initially, the role of *α*AR-agonists and GCs in this condition was investigated utilizing *in vivo* animal models of trauma-induced stridor [109]. In these experiments, a ferret model of postintubation croup was used to illustrate that both the topical application of the selective *α*AR-agonist, oxymetazoline, and the intramuscular administration of dexamethasone completely ablated the subglottic edema induced by traumatic intubation [109]. Subsequent studies have further supported the use of these therapies for the management of postextubation stridor in adults and children [110–119]. In concordance with the beneficial effects of nebulized epinephrine and dexamethasone in the treatment of stridor from viral croup [87], most clinical studies have focused on evaluating the effectiveness of these medications in the prevention and treatment of postextubation stridor. 

Racemic epinephrine and L-epinephrine have been shown in RCTs to improve clinical scores in pediatric patients who developed stridor in the immediate postextubation period [110]. As expected, this *α*AR-mediated effect is short lasting [110], which mandates the need for alternative or coadjuvant therapies. Accordingly, the efficacy of GCs in the prevention of postextubation stridor has also been extensively studied, especially when comparing them to the use of *α*AR-agonists alone. Among high-risk neonates with a history of traumatic, multiple, or prolonged intubation, the use of either one or three doses of intravenous (i.v.) dexamethasone (0.25 mg/kg/dose) resulted in fewer patients with postextubation stridor and fewer patients requiring reintubation [111, 112]. Similar findings have been reported when treating pediatric patients outside of the neonatal period. A prospective RCT of children less than 5 years of age, who were intubated and mechanically ventilated for >48 hrs, reported that IV dexamethasone (0.5 mg/kg/dose 6–12 hours before the extubation and then every 6 hours for a total of 6 doses) was associated with lower croup scores, less frequency of stridor, and fewer episodes of reintubation and/or acute treatment with nebulized epinephrine [113]. Prednisolone (1 mg/kg/dose) has also been identified to reduce the duration of intubation and the need for reintubation in children >6 months old intubated for croup [114]. However, a separate RCT showed no benefit of GC in preventing reintubation following an initial “failed” extubation due to stridor [120]. Importantly, children that required reintubation in this study had significant neurologic impairment, which correlated closely with reintubation rates [120]. Recently, the utility of GC in the treatment of postintubation stridor has been summarized in a cochrane review of 11 RCTs (2 neonatal RCTs and 3 RTCs in older children) that pooled a total of 2301 patients, adult and pediatric combined. The authors concluded that while there is insufficient evidence supporting routine GC use for the prevention of postextubation stridor in children, there is a consistent trend towards reduced rates of reintubation and postextubation stridor, particularly among high-risk patients [121]. Of note, the analysis also identified that GC significantly reduced postextubation stridor in adults, although GC-induced effect on reintubation rates did not reach statistical significance [121]. 

In summary, there is compelling evidence demonstrating beneficial effects of using both nebulized epinephrine and systemic GC to reduce the upper airway intraluminal narrowing induced by endotracheal intubation (Table 4). These data are consistent with previous clinical studies that established the efficacy of *α*AR-agonists and GCs in treating stridor during acute viral laryngotracheobronchitis [57, 87]. Notwithstanding this evidence, the clinical usefulness of these therapies at improving critical outcomes (i.e., reintubation rates) requires further investigation, especially because these effects have not yet been observed in all groups of pediatric patients. Thus, GC use during the postextubation period requires careful evaluation of the underlying disorder and associated comorbidities in neonatal and pediatric patients.

### 5.4. Safety of *α*AR-Agonists and GCs during the Therapy of Stridor in Children

The known potential adverse effects of *α*AR-agonists and GC administration in children requires consideration. GCs use predisposes to many systemic side effects including hyperglycemia, decreased bone density, altered hypothalamic-pituitary-adrenal axis, and abnormal immune responses [122]. Specific to the pediatric population is the potential decreased rate of growth [123] associated with the prolonged use of systemic GCs and high-doses of inhaled GCs [124]. A specific concern regarding the use of GC in viral-induced croup is the potential generation of severe bacterial [125] or fungal laryngotracheitis [126], which are reported complications in this setting. With regards to *α*AR-agonists, nebulized racemic epinephrine has been associated with potential serious cardiovascular complications in children treated for viral croup [90]. These effects are related to its nonselective *α*AR and *β*AR actions, and their action may induce tachycardia, hypertension, and myocardial infarction [90]. Despite these potential serious complications, the vast majority of clinical studies have demonstrated that both GCs and nebulized epinephrine are well tolerated without serious complications consistently reported during the therapy of pediatric stridor [57, 87].

### 5.5. Potential Clinical Synergism of *α*AR-Agonists and GCs in Pediatric Stridor

As previously mentioned, tachyphylaxis and rebound may be deleterious consequential effects of *α*AR-agonists. The latter side effects are present in the nasal mucosa [59–62] and more distal segments of the upper airway [64]. These changes in *α*AR function are attributed to AR-homologous desensitization, which is modulated by GC signaling [29]. This molecular mechanism provides the basic foundation for the potential synergistic use of combined GC and *α*AR-agonist therapy. Two recent RCTs have demonstrated that GCs actually prevent *α*AR-agonist induced tachyphylaxis and rebound in the treatment of nasal congestion [78, 79]. The beneficial effects of the combined use of GCs and nebulized epinephrine in the treatment of respiratory distress associated with acute viral illnesses in children have also been reported [80]. Although these studies are often aimed to assess specific respiratory syndromes, such as viral croup or viral bronchiolitis, the effect of nebulized epinephrine is likely due to its *α*AR-agonist action in the upper airway, regardless of underlying syndrome [91]. The synergy of the use of GCs and nebulized epinephrine was supported by a recent multicenter RCT involving 800 infants with viral bronchiolitis [80]. They convincingly demonstrated that the combined use of dexamethasone and nebulized epinephrine provided a significant reduction in hospital admission rate and improved clinical respiratory distress scores superior to either therapy alone [80]. This seminal RCT suggests a significant synergism between epinephrine and dexamethasone, particularly because dexamethasone alone has not been associated with significant clinical benefit in viral bronchiolitis, as demonstrated by several RCTs [127, 128]. In the setting of viral-induced croup, the addition of dexamethasone to epinephrine prevents relapses and hospital admissions [129], as well as improves croup scores [130–132]. Nonetheless, until clinical evidence from RCTs specifically designed to investigate the synergism of *α*AR-agonist and GCs in pediatric stridor is available, combined therapy is not currently recommended for the routine treatment of stridor in children.

## 6. Conclusions and Future Directions

There is compelling scientific evidence supporting the use of alpha-adrenergic (*α*AR) agonists and glucocorticosteroids (GCs) in the treatment of pediatric upper airway obstruction and stridor. At the “bench” level, it is clear that the regulation of the vasomotor tone and extravascular volume in the upper airway mucosa is the result of complex mechanisms and interactions between *α*AR signaling and genomic and nongenomic GC effects. The latter molecular and cellular events translate into better airflow mechanics, as observed in several human and nonhuman upper airway physiological models. When tested in the clinical arena, *α*AR-agonists and GCs hold their beneficial effects in upper airway function. Several randomized controlled trials (RCTs) have consistently demonstrated that GC and *α*AR-agonists, particularly dexamethasone and nebulized epinephrine, improve respiratory distress scores and reduce need for further medical care in children with viral-induced croup or traumatic postextubation stridor. Future research directions should include clinical and translational studies that investigate the potential benefits of selective *α*AR-agonists and combined therapy (GC and *α*AR-agonists) in the treatment of upper airway obstruction and stridor in neonates, infants, and children.

## Figures and Tables

**Table 1:** Molecular and cellular effects of *α*AR-agonists and glucocorticoids in the upper airway.

Treatment modality	Physiological effects
*α*AR-agonists	(i) Increased vascular smooth muscle (VSM) contractile tone
	Gq protein-mediated VSM contraction (*α*1)
	Gi protein-mediated VSM impaired relaxation (*α*2)
	(ii) Modulation of mucosa blood flow and thickness
	(iii) Increased nasal cavity cross-sectional area
	(iv) Increased oropharyngeal patency
	(v) Increased subglottic intraluminal diameter

Glucocorticoids	(i) Transrepression of AP-1 and NF-*κ*B transcription factors
	(ii) Regulation of mitogen-activated protein kinases (MAPKs)
	(iii) Modulation of vascular endothelial growth factor (VEGF) expression
	(iv) Enhanced *α*AR-signaling and prevention of *α*AR-homologous desensitization
	(v) Modulation of airway mucosa inflammation
	(vi) Reduced airway mucosa thickness
	(vii) Increased nasal and subglottic patency

**Table 2:** Effects of *α*AR-agonists and glucocorticoids in upper airway breathing mechanics.

Treatment modality	Physiological effects
*α*AR-agonists	(i) Increased nasal inspiratory flow (PNIF)
	(ii) Decreased oropharyngeal resistance and collapsibility
	(iii) Decreased total respiratory resistance (*R*_*T*_)
	(iv) Decreased inspiratory and expiratory resistance (*R *_AW0.5I_ and *R *_AW0.5E_)
	(v) Decreased work of breathing (WOB)
	(vi) Paradoxical nasal obstruction (vasomotor rebound)
	(vii) Rebound increase in total respiratory resistance (*R*_*T*_)

Glucocorticoids	(i) Increased nasal inspiratory flow (PNIF)
	(ii) Decreased nasal airway resistance (NAR)
	(iii) Increased nasal volume by acoustic rhinometry (AR)
	(iv) *α*AR-induced vasomotor rebound prevention (changes in PNIF, AR and NAR)

**Table 3:** Clinical effects of *α*AR-agonists and glucocorticoids in viral-induced stridor.

Treatment modality	Clinical outcomes
*α*AR-agonists	(i) Transient improvements in croup symptom scores compared to placebo at 10 and 30 minutes after administration (duration 120 minutes)
(i) Nebulized racemic epinephrine (ii) Nebulized (L) isomeric epinephrine	(ii) Shorter hospital stay

Glucocorticoids	
(i) Dexamethasone (0.15–0.6 mg/kg)*	(i) Significant improvement in croup symptom scores, starting about an hour after administration
(ii) Nebulized budesonide (2–4 mg)	(ii) Sustained effect in croup symptom scores (peak 6–12 hours after administration)
(iii) Betamethasone (0.4 mg/kg/dose)	(iii) Decreased number of return visits or (re)admissions
(iv) Prednisolone (1 mg/kg/dose)	(iv) Decreased length of time spent in the hospital

^∗^The majority of large randomized clinical trials have been conducted with dexamethasone, but there is clinical evidence suggesting equivalent responses with other glucocorticoids used in viral-induced stridor.

**Table 4:** Clinical effects of *α*AR-agonists and glucocorticoids in postextubation stridor.

Treatment modality	Clinical outcomes
*α*AR-agonists: (i) Nebulized racemic epinephrine (ii) Nebulized (L) isomeric epinephrine	(i) Transient improvements in croup symptom scores compared to placebo at 10 and 30 minutes after administration (duration 120 minutes)

Glucocorticoids	(i) Reduced rates of postextubation stridor in adults and certain pediatric groups (i.e., high-risk patients)
(i) Dexamethasone (0.15–0.6 mg/kg)	(ii) Probable decrease in reintubation rates in select cases, but not in general adult and pediatric populations
